# Superoxide Anions in Paraventricular Nucleus Modulate Adipose Afferent Reflex and Sympathetic Activity in Rats

**DOI:** 10.1371/journal.pone.0083771

**Published:** 2013-12-23

**Authors:** Lei Ding, Ling-Li Zhang, Run Gao, Dan Chen, Jue-Jin Wang, Xing-Ya Gao, Yu-Ming Kang, Guo-Qing Zhu

**Affiliations:** 1 Key Laboratory of Cardiovascular Disease and Molecular Intervention, Department of Physiology, Nanjing Medical University, Nanjing, Jiangsu, China; 2 Department of Physiology and Pathophysiology, Cardiovascular Research Center, Xi'an Jiaotong University School of Medicine, Xi'an, Shanxi, China; Hosptial Infantil Universitario Niño Jesús, CIBEROBN, Spain

## Abstract

**Background:**

Adipose afferent reflex (AAR) is a sympatho-excitatory reflex induced by chemical stimulation of white adipose tissue (WAT). Ionotropic glutamate receptors including NMDA receptors (NMDAR) and non-NMDA receptors (non-NMDAR) in paraventricular nucleus (PVN) mediate the AAR. Enhanced AAR contributes to sympathetic activation and hypertension in obesity rats. This study was designed to investigate the role and mechanism of superoxide anions in PVN in modulating the AAR.

**Methodology/Principal Findings:**

Renal sympathetic nerve activity (RSNA) and mean arterial pressure (MAP) were recorded in anesthetized rats. AAR was evaluated by the RSNA and MAP responses to injections of capsaicin into four sites of right inguinal WAT (8.0 nmol in 8.0 µl for each site). Microinjection of polyethylene glycol-superoxide dismutase (PEG-SOD), the superoxide anion scavenger tempol or the NAD(P)H oxidase inhibitor apocynin into the PVN decreased the baseline RSNA and MAP, and attenuated the AAR. Unilateral WAT injection of capsaicin increased superoxide anions in bilateral PVN, which was prevented by the WAT denervation. WAT injection of capsaicin increased superoxide anion level and NAD(P)H oxidase activity in the PVN, which was abolished by the PVN pretreatment with the combined NMDAR antagonist AP5 and non-NMDAR antagonist CNQX. Microinjection of the NMDAR agonist NMDA or the non-NMDAR agonist AMPA increased superoxide anion level and NAD(P)H oxidase activity in the PVN.

**Conclusions:**

NAD(P)H oxidase-derived superoxide anions in the PVN contributes to the tonic modulation of AAR. Activation of ionotropic glutamate receptors in the PVN is involved in the AAR-induced production of superoxide anions in the PVN.

## Introduction

It is known that white adipose tissues (WAT) are innervated by both sensory and efferent sympathetic fibers [1], [29]. WAT injection of leptin increased sympathetic outflow to epididymal WAT [21], brown adipose tissue (BAT), adrenal medulla, pancreas and liver [22], and kidney [31] in rats. We found that the sympatho-excitatory reflex, adipose afferent reflex (AAR), were induced by several chemicals such as capsaicin, bradykinin, adenosine or leptin in the WAT [28]. One of the physiological significances of the AAR is to increase sympathetic outflow, and thus to promote energy expenditure and lipolysis [1], [34]. More recently, we found that the AAR induced by visceral WAT stimulation was enhanced in obesity and obesity-related hypertension rats, and the enhanced AAR contributed to sympathetic activation in obesity hypertension [33]. The AAR study offers direct measurements of tonic sympathoexcitation originating from the WAT, and visceral fat is a potentially treatable candidate for one possible source of increased sympathetic outflow [8].

Paraventricular nucleus (PVN) of the hypothalamus is an integrative site in the control of sympathetic outflow and cardiovascular activity [6]. We found that PVN lesion with kainic acid abolished the AAR in normal rats [28]. Inhibition of PVN neurons with lidocaine abolished the AAR, attenuated sympathetic activity and hypertension in obesity hypertensive rat, and chemical stimulation of iWAT caused more c-fos expression in the PVN in obesity hypertension rats than that in control rats [33]. These results indicate that PVN plays an important role in the control of AAR. Blockade of AAR may have beneficial effects on attenuating obesity hypertension.

Oxidative stress in sympathetic premotor neurons including PVN and rostral ventrolateral medulla (RVLM) contributes to sympathetic activation in renovascular hypertension [24]. Increased superoxide anions in the PVN are involved in the enhanced cardiac sympathetic afferent reflex (CSAR) and renal sympathetic nerve activity (RSNA) in renovascular hypertension [13]. NAD(P)H oxidase in the PVN contributes to elevated sympathetic activity and the hypertensivogenic actions induced by mineralocorticoid excess [35]. We found that superoxide dismutase 1 (SOD1) gene transfer into the PVN attenuates sympathetic activity and hypertension in spontaneously hypertensive rats [38], and improves post-infarct myocardial remodeling and ventricular function in chronic heart failure rats [12]. The first aim of the present study was designed to determine whether superoxide anions in the PVN are involved in modulating the AAR. Recently, we found that bilateral PVN microinjection of NMDA receptor (NMDAR) antagonist AP5, or non-NMDAR antagonist CNQX attenuated the AAR, and combined AP5 and CNQX abolished the AAR, indicating ionotropic glutamate receptors in the PVN mediate the AAR [7]. The second aim of the present study was to determine whether the activation of ionotropic glutamate receptors in the PVN is involved in the AAR-induced increases in superoxide anions in the PVN.

## Materials and Methods

Experiments were carried out on 132 male Sprague–Dawley rats weighing between 350 and 400 g. which were approved by the Experimental Animal Care and Use Committee of Nanjing Medical University and complied with the Guide for the Care and Use of Laboratory Animals (NIH Publication No. 85-23, revised 1996). The rats were caged in a controlled temperature and humidity with a 12-hour light/dark cycle. Standard laboratory chow and drinking water were available ad libitum. The rat was anesthetized with intraperitoneal injection of urethane (800 mg/kg,) and α-chloralose (40 mg/kg). Supplemental doses of anaesthetics were administered intravenously to maintain an adequate depth of anesthesia during the experiment. The rat was ventilated with room air using a rodent ventilator (683, Harvard Apparatus Inc, USA). The right carotid artery was cannulated for recording of mean arterial pressure (MAP). The rats were allowed to stabilize for 30–60 min after surgery.

### RSNA recording

Left renal nerve was isolated and cut distally to eliminate its afferent activity through a retroperitoneal incision. The central end of the nerve was placed on a pair of silver electrodes and immersed in warm mineral oil. The signals were amplified with a four-channel AC/DC differential amplifier (DP-304, Warner Instruments, Hamden, CT, USA) with a high pass filter at 10 Hz and a low pass filter at 3,000 Hz. The RSNA was integrated at a time constant of 100 ms. Background noise was measured after section of the central end of the nerve at the end of the experiment and was subtracted from the integrated values of the RSNA [10], [11]. RSNA and MAP were simultaneously recorded on a PowerLab data acquisition system (8/35, ADInstruments, Castle Hill, Australia).

### Evaluation of AAR

Capsaicin is a valuable tool for studying the function of afferent fiber via activating transient receptor potential vanilloid 1 (TRPV1) in sensory fibers [15], [18]. Endogenous capsaicin-like substances which activate TRPV1 have been identified [16], [39]. AAR induced by WAT injection of capsaicin has been reported recently [7], [28], [33]. Briefly, right inguinal WAT (iWAT) was exposed through an inguinal area incision. Four thin and sharp stainless steel tubes (0.31 mm outer diameter) were inserted into the fat pad 3 mm below the surface of the fat pads. The tips of these tubes were 4 mm apart from each other and were connected with a 4-channel programmable pressure injector (PM2000B, MicroData Instrument, NJ, USA). The AAR was induced by the injections of capsaicin (1.0 nmol µl^−1^) into four sites of the right iWAT at a rate of 4.0 µl min^−1^ for 2 min for each site. AAR was evaluated by the RSNA and MAP responses to injections of capsaicin. At the end of the experiment, the same volume of Evans blue was injected into the iWAT. Histological identification of the WAT was made 30 min later. The dye was localized in the WAT and the diffusion diameter was less than 3 mm in all rats.

### PVN microinjection

**Figure 1:**
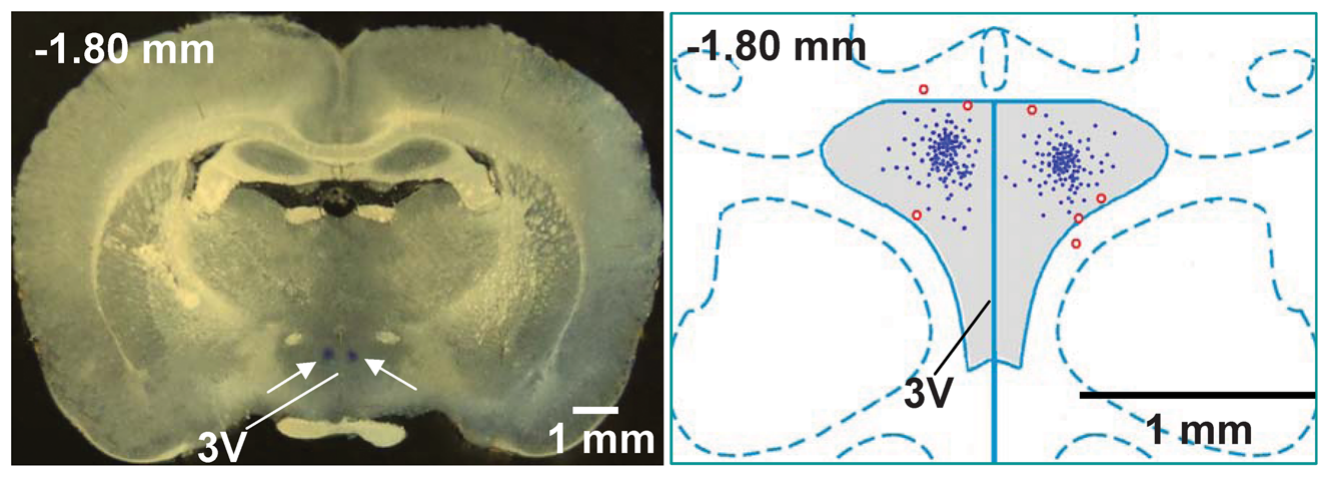
Microinjection sites in PVN area. **Left panel, a representative photo of microinjection sites in the PVN evaluated by 50**. Arrows show the microinjection sites; Right panel, a schematic representation of microinjection sites. Blue dots or red open circles represent the sites of termination of the microinjections. Blue dots are considered to be within the PVN. Red open circles are considered to be outside of the PVN or at the margin of the PVN, which were excluded for data analysis. 3V, the third ventricle.

Stereotaxic coordinates for PVN were 1.8 mm caudal from bregma, 0.4 mm lateral to the midline and 7.9 mm ventral to the dorsal surface. Microinjection volume for each side of the PVN was 50 nl, and the bilateral PVN microinjections were completed within 1 min. At the end of the experiment, 50 nl of Evans blue was injected into each microinjection site for histological identification of the microinjection sites [14], [30]. Total 7 rats scattered in different groups were excluded from data analysis because the microinjection sites were outside one side of the PVN (Figure 1).

### Measurement of superoxide anion level and NAD(P)H oxidase activity

Rats were euthanized with an overdose of pentobarbital sodium (200 mg/kg, iv). Brain was removed and flash-frozen in liquid nitrogen and stored at −70°C until being sectioned. A 450-µm-thick coronal section was cut through the hypothalamus and incorporated the PVN areas. Then, the PVN areas were punched out with a 15-gauge needle (inner diameter 1.5 mm), homogenized and centrifuged in lysis buffer. Total protein concentration in the homogenate was measured with the Bradford assay (BCA; Pierce, Santa Cruz, CA, USA). Superoxide anion level and NAD(P)H oxidase activity in the PVN were measured with lucigenin-derived chemiluminescence method as we previously reported [13], [26], [27]. Briefly, the photon emission was started by adding dark-adapted lucigenin for determining the superoxide anion level, by adding both NAD(P)H and dark-adapted lucigenin for determining the NAD(P)H oxidase activity. Light emission was measured for 10 times in 10 min with a luminometer (20/20n, Turner, BioSystems, Sunnyvale, USA). The values were averaged, and expressed as mean light unit (MLU) per minute per milligram of protein.

### *In situ* detection of superoxide anions

Specific fluorogenic probe dihydroethidim (DHE) was used to detect in situ superoxide anions in the PVN as we previously reported [27]. The rats were euthanized with an overdose of pentobarbital sodium (200 mg/kg, iv). Brains were quickly removed, frozen with liquid nitrogen, embedded into OCT, and cryostat sectioned (30 µm, coronal) onto chilled microscope slides. Then the sections were thawed at room temperature, rehydrated with phosphate-buffered saline, and incubated for 5 minutes in the dark with DHE (1 µmol/L). After washing with phosphate-buffered saline, the DHE fluorescence in the coronal sections was visualized under a fluorescence microscope (BX51, Olympus, Tokyo, Japan). Images were collected by using an Olympus BX51 microscope coupled with an Olympus DP70 digital camera at ×100. Detector and laser settings were kept constant among all samples within individual experiment. The control and experimental samples were always processed in parallel. Fluorescence intensity was analyzed and quantified with Image-Pro Plus 6.0 by using the same parameters.

### Surgical denervation of iWAT

The right iWAT was isolated without damaging the vessels. A drop of 1% toluidine blue was applied to the fat pad to facilitate visualization of the nerves. All nerve fibers that were visible in these areas were cut [9]. Vessels connected with the iWAT were painted with 10% phenol solution to destroy any remaining nerve fibers in this area. Effectiveness of this method has been identified by the greatly decreased SP level (a sensory nerve marker) and NE level (a sympathetic nerve marker) in the denervated iWAT [28].

### Drugs

PEG-SOD, tempol, apocynin, capsaicin, D-2-amino-5-phosphonovaleric acid (AP5), 6-cyano-7-nitroquinoxaline-2,3-dione (CNQX), N-methyl-d-aspartate (NMDA), alpha-amino-3-hydroxy-5-methyl-4-isoxazole propionic acid (AMPA), DMSO, NAD(P)H and lucigenin, were obtained from Sigma Chemical Co. Capsaicin stock solution was dissolved in absolute ethanol and was diluted before injection to a final concentration of 1% of the stock solution, 1% of Tween 80 and 98% of Saline. The vehicle was used as control. Apocynin was dissolved in normal saline containing 1% of DMSO, and the same concentration of DMSO was used as control. All other chemicals were dissolved in normal saline.

### Experimental design

#### Experiment 1

The roles of superoxide anions and NAD(P)H oxidase in the modulation of AAR were determined. The rats were randomly divided into 7 groups (n = 6 for each group), which were subjected to the PVN microinjection of saline, three doses of PEG-SOD (0.2, 1 or 5 units), superoxide anion scavenger tempol (20 nmol), 1% of DMSO or NAD(P)H oxidase inhibitor apocynin (1 nmol). AAR was induced 8 min after the PVN microinjection.

#### Experiment 2

The effects of iWAT injection of capsaicin on the superoxide anion level and NAD(P)H oxidase activity in the PVN were determination. The rats were randomly divided into 5 groups (n = 6 for each group). Three groups of them were used for in situ detection of superoxide anions with DHE fluorescence in the PVN, and were subjected to the right iWAT injection of saline or capsaicin, or capsaicin pretreated with the right iWAT denervation. Four sections were used for analysis for each rat. Other two groups of rats were subjected to the right iWAT injection of saline or capsaicin, and were used for the measurements of superoxide anion levels and NAD(P)H oxidase activity in the PVN with chemiluminescence method. The rats were euthanized 15 min after the iWAT injection of saline or capsaicin for the measurements.

#### Experiment 3

Based on our recent findings that both NMDAR and non-NMDAR in the PVN mediate AAR [7], the experiments were designed to determine whether superoxide anions and NAD(P)H oxidase activity in the PVN are related to NMDAR and non-NMDAR in the PVN. The rats were randomly divided into 10 groups (n = 6 for each group). Six groups of them were subjected to the PVN microinjection of saline, NMDAR agonist NMDA (9 nmol), non-NMDAR agonist AMPA (9 nmol), NMDAR antagonist AP5 (9 nmol), non-NMDAR antagonist CNQX (9 nmol), or combined AP5 (9 nmol) and CNQX (9 nmol). The rats were euthanized 15 min after the microinjection for the measurements of superoxide anion levels and NAD(P)H oxidase activity in the PVN. Other four groups of rats were subjected to the iWAT injection of capsaicin pretreated with saline, AP5 (9 nmol), CNQX (9 nmol), or combined AP5 (9 nmol) and CNQX (9 nmol). The pretreatment was carried out 5 min before the injection of capsaicin. The rats were euthanized 15 min after the iWAT injection of capsaicin for the measurements.

### Statistics

Baseline RSNA or MAP were determined by averaging 2 min of its maximal responses within the time range of time from 3 min to 7 min after the PVN microinjection. AAR was evaluated by averaging 2 min of the maximal RSNA and MAP responses to capsaicin within the time range from 10 min to 15 min after the iWAT injection of capsaicin. Comparisons between two observations in the same animal were assessed by Student's paired t test. The differences between groups were determined with a two-way ANOVA followed by the Newman-Keuls test for post hoc analysis of significance. All data were expressed as mean ± SE. A value of P<0.05 was considered statistically significant.

## Results

### Effects of different doses of PEG-SOD

**Figure 2:**
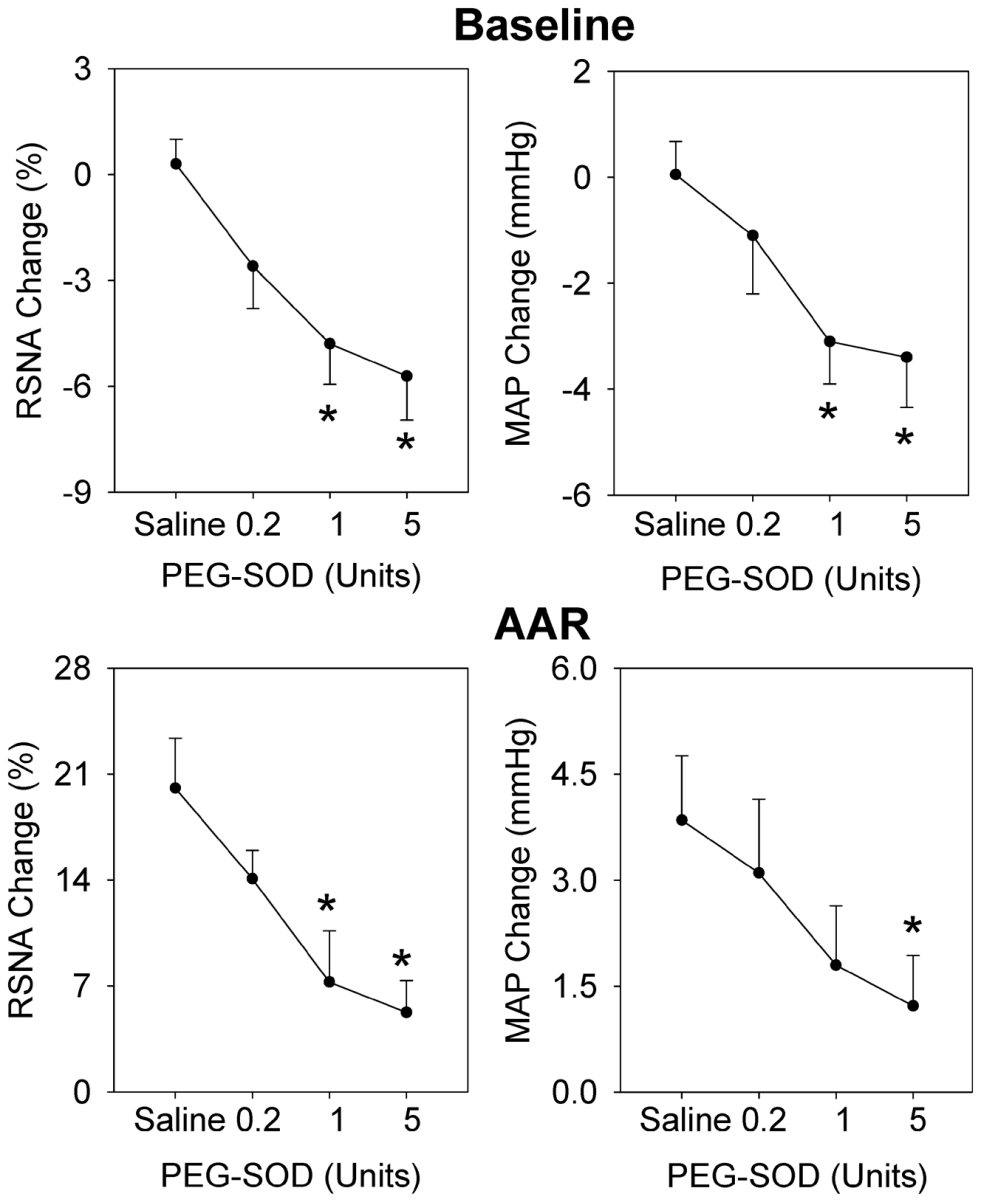
Effects of PVN pretreatment with saline or different doses of PEG-SOD (0.2, 1 or 5 units) on the baseline RSNA and MAP, and the AAR induced by capsaicin in the right iWAT. Capsaicin was administered 8±SE. n = 6 for each group. *P<0.05 vs. Saline.

**Figure 3:**
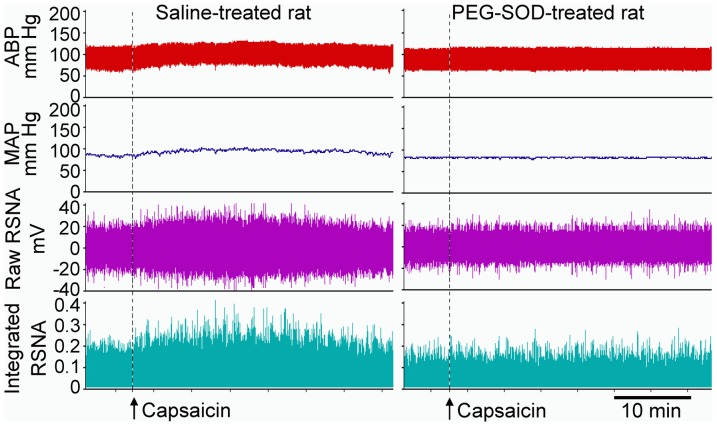
Representative traces showing that PVN pretreatment with PEG-SOD (5 units) attenuated the AAR induced by the iWAT injection of capsaicin. Capsaicin was administered 8

PVN microinjection of moderate or high dose of PEG-SOD (1 or 5 units) to scavenge superoxide anions in the PVN significantly decreased baseline RSNA and MAP, and attenuated the capsaicin-induced AAR (Figure 2). Two-way ANOVA revealed no significant interaction between the iWAT injection of capsaicin and the PVN microinjection of PEG-SOD on the RSNA (F (3,40) = 1.615, *P* = 0.201) or the MAP (F (3,40) = 0.077, *P* = 0.972), but very significant main effects for the capsaicin (RSNA: F(1,40) = 83.929, *P*<0.001; MAP: F(1,40) = 40.147, *P*<0.001) and the PEG-SOD (RSNA: F (3,40) = 8.42, *P*<0.001; MAP: F (3,40) = 4.274, P = 0.01), indicating that these factors acted independently. High dose of PEG-SOD reduced the capsaicin-induced RSNA change from 20.1±3.3 to 5.3±2.1% (*P*<0.01), and MAP change from 3.9±0.9 to 1.1±0.6 mmHg (*P*<0.05). Representative AAR recordings showed that iWAT injection of capsaicin increased RSNA and MAP in the saline-treated rat, but failed to cause obvious changes in the RSNA and MAP in the PEG-SOD-treated rat (Figure 3).

### Effects of tempol and apocynin

**Figure 4:**
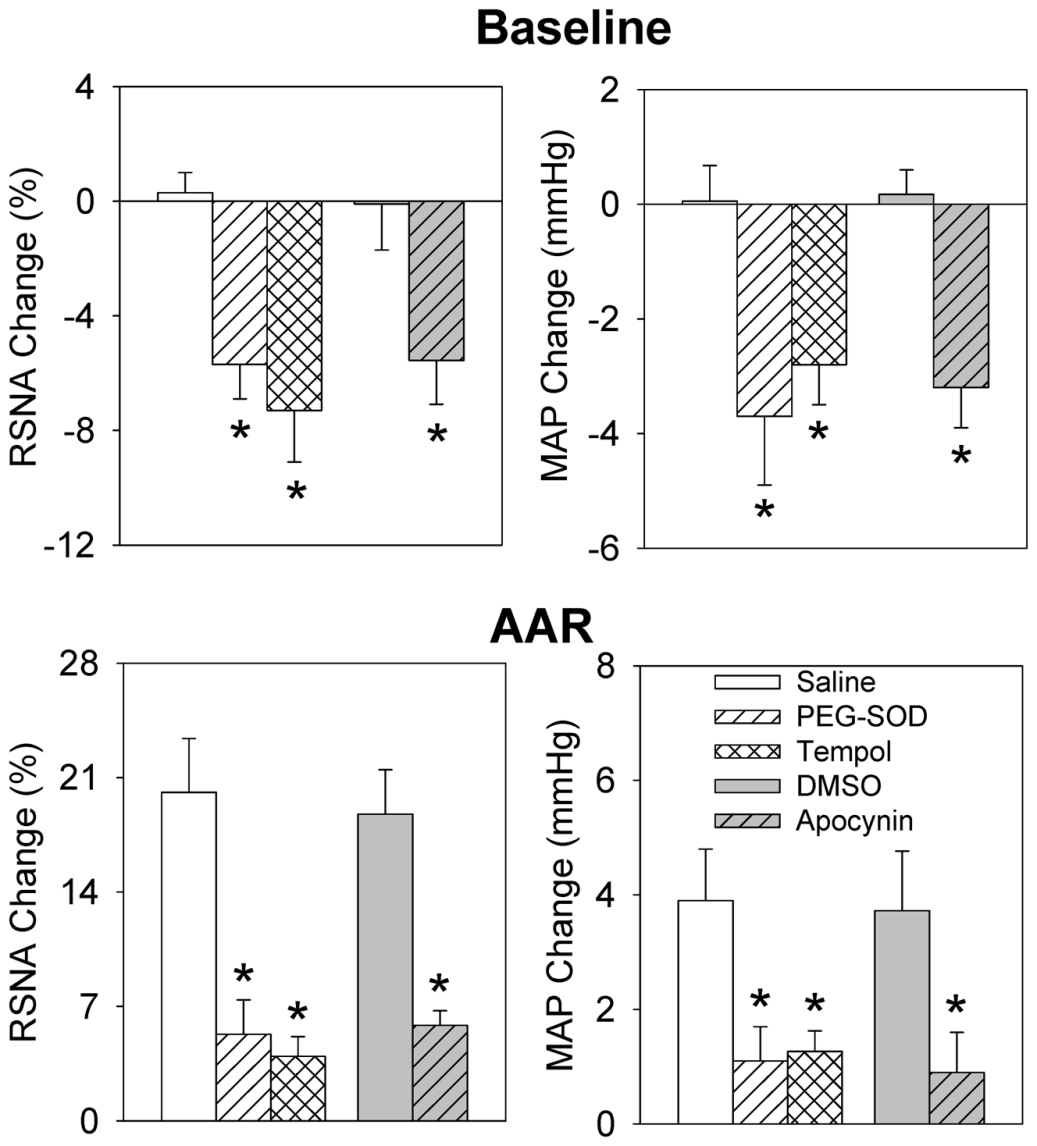
Effects of PVN pretreatment with saline, PEG-SOD (5 units), superoxide anion scavenger tempol (20 nmol), 1% DMSO or NAD(P)H oxidase apocynin (1 nmol) on the baseline RSNA and MAP, and the AAR induced by capsaicin in the right iWAT. Capsaicin was administered 8±SE. n = 6 for each group. *P<0.05 vs. Saline or DMSO.

Microinjections of superoxide anion scavenger tempol or NAD(P)H oxidase inhibitor apocynin into the PVN decreased the baseline RSNA and MAP, and attenuated the capsaicin-induced AAR (Figure 4). Two-way ANOVA did not reveal a significant interaction between the iWAT injection of capsaicin and the PVN microinjections of tempol or apocynin on the RSNA or MAP (RSNA: F (3,40) = 2.476, *P* = 0.075; MAP: F (3,40) = 0.121, *P* = 0.947). Instead, main effects were confirmed for the iWAT injection of capsaicin (RSNA: F (1,40) = 107.276, *P*<0.001; MAP: F(1,40) = 49.172, *P*<0.001) and microinjections of tempol or apocynin (RSNA: F (3,40)  = 17.16, *P*<0.001; MAP: F (3,40) = 9.119, *P*<0.001). Apocynin reduced the capsaicin-induced RSNA change from 18.8±2.7 to 5.8±0.9% (*P*<0.01), and MAP change from 4.0±1.1 to 0.9±0.7 mmHg (*P*<0.05).

### *In situ* detection of superoxide anions

**Figure 5:**
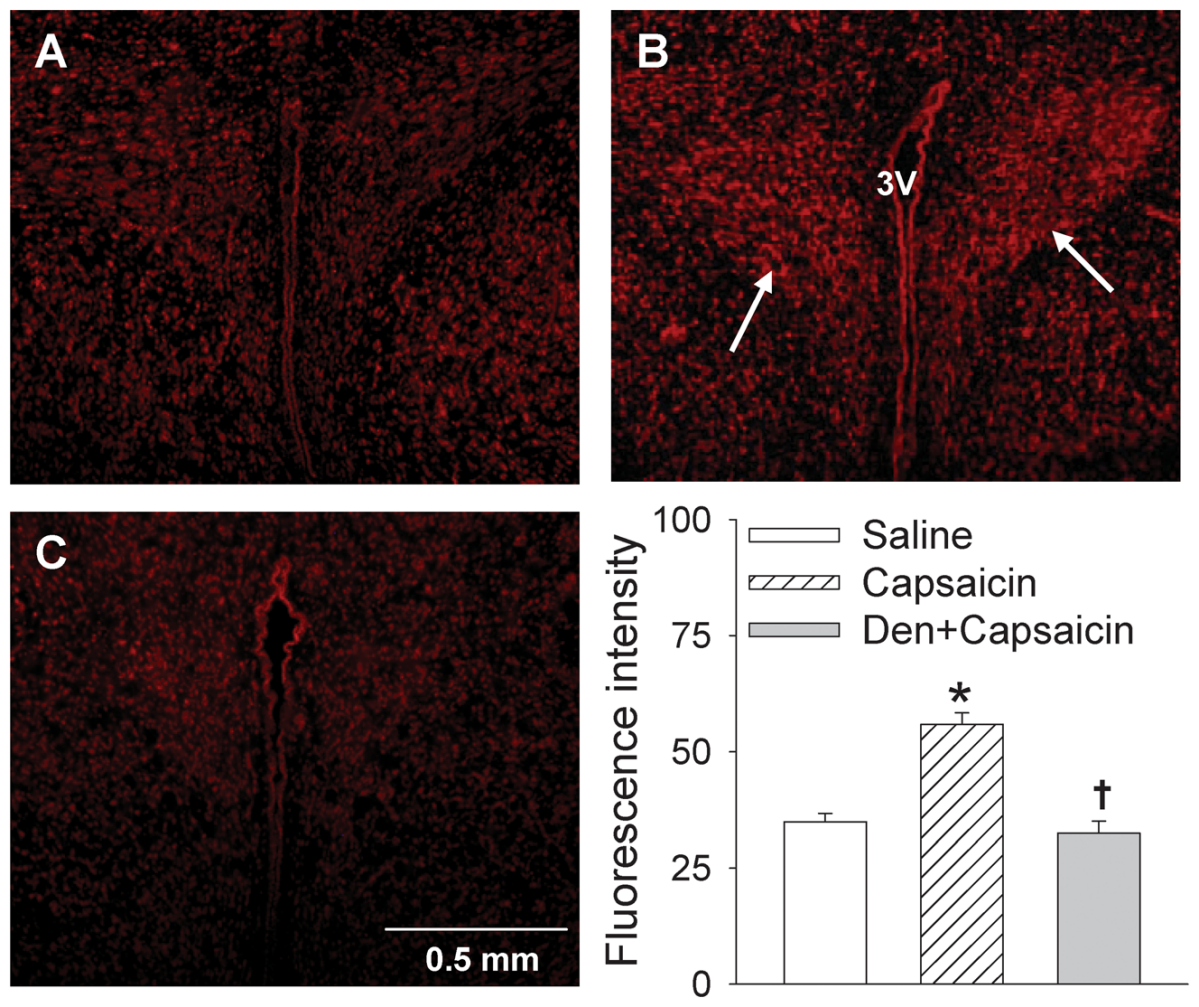
Effects of right iWAT injection of saline or capsaicin on the superoxide anion level in the PVN, indicated by DHE fluorescence intensity. 3V, the third ventricle. A, iWAT injection of saline; B, iWAT injection of capsaicin. Arrows points the PVN areas. C, iWAT injection of capsaicin in the rat treated with iWAT denervation (Den). Values are mean±SE. *P<0.05 vs. Saline. † P<0.05 vs. capsaicin only. n = 6 for each group.

Injection of capsaicin into the right iWAT increased the superoxide anions in the bilateral PVN compared with injection of saline. The relative fluorescent intensity in the PVN was 34.9±1.8 in saline-treated rats and 55.9±2.5 in capsaicin-treated rats (P<0.01). In the right iWAT denervated rats, capsaicin failed to cause any change in superoxide anions in the PVN (Figure 5).

### Effects of injection of capsaicin in iWAT

**Figure 6:**
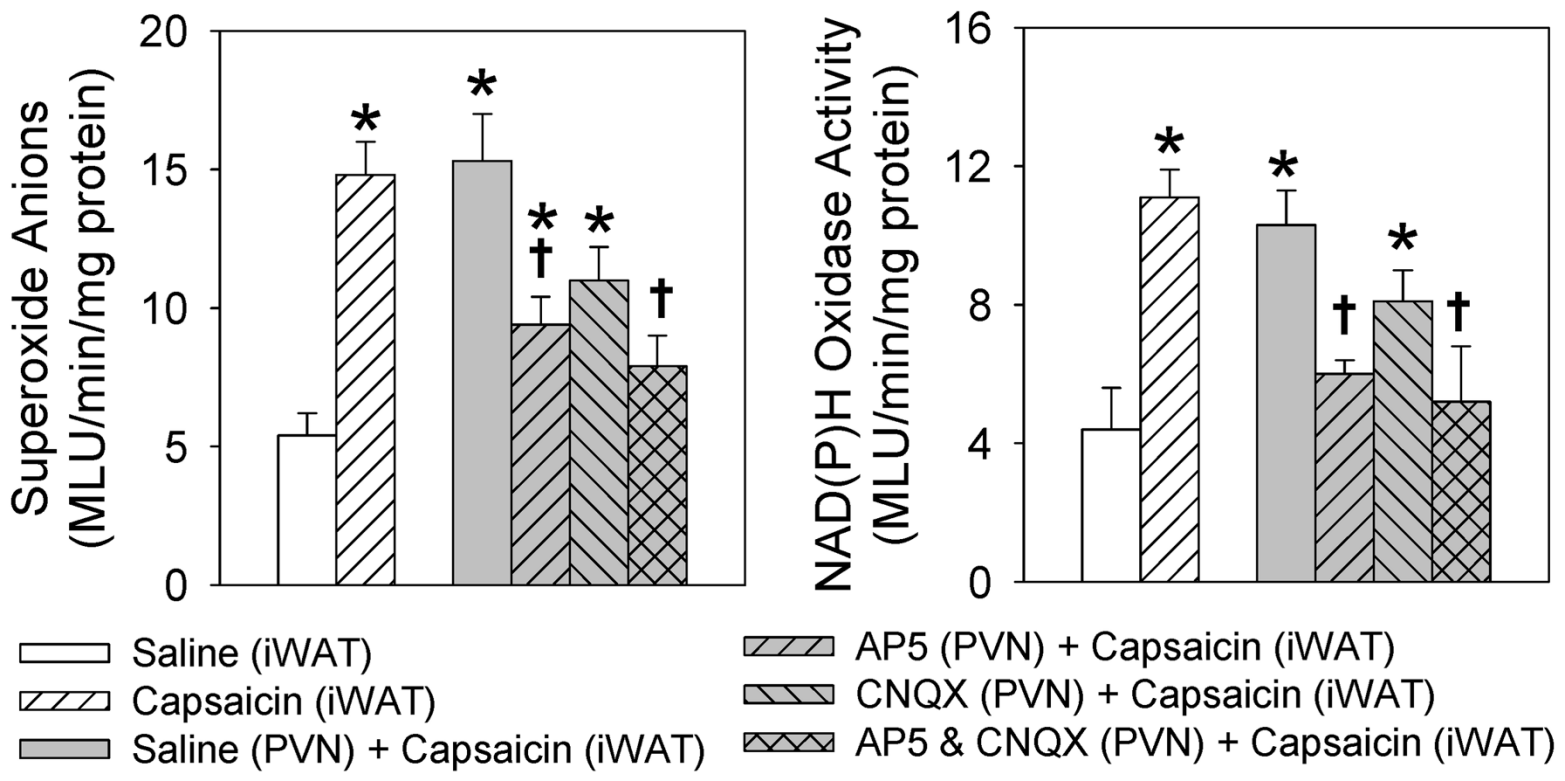
Effects of administration of saline (iWAT), capsaicin (iWAT), saline (PVN) + capsaicin (iWAT), AP5 (PVN) + capsaicin (iWAT), CNQX (PVN) + capsaicin (iWAT) or AP5 & CNQX (PVN) + capsaicin (iWAT) on superoxide anion level and NAD(P)H oxidase activity in the PVN. The measurements were made 15±SE. n = 6 for each group. *P<0.05 vs. Saline (iWAT); † P<0.05 vs. Saline (PVN) + capsaicin (iWAT).

Two-way ANOVA revealed no significant interaction between the iWAT injection of capsaicin and the PVN microinjection of ionotropic glutamate receptors agonists or antagonists on the superoxide anion level (F (3,60) = 2.617, *P* = 0.059) or the NAD(P)H oxidase activity (F (3,60) = 0.952, *P* = 0.421), but very significant main effects for the capsaicin (superoxide anion: F(2,60) = 48.949, *P*<0.001; NAD(P)H oxidase: F(2,60) = 23.936, *P*<0.001) and the PVN microinjection (superoxide anion: F (6,60) = 16.944, *P*<0.001; NAD(P)H oxidase: F (6,60) = 9.630, *P*<0.001), indicating that these factors acted independently. Compared with saline, injection of capsaicin into the right iWAT increased the superoxide anion level (14.8±1.2 vs. 5.4±0.8 MLU/min/mg protein, *P*<0.001) and NAD(P)H oxidase activity (11.1±0.8 vs. 4.4±1.2 MLU/min/mg protein, *P*<0.01) in the PVN. Microinjection of combined NMDAR antagonist AP5 and non-NMDAR antagonist CNQX into the PVN prevented the increases in superoxide anion level (15.3±1.7 vs. 7.9±1.1 MLU/min/mg protein, *P*<0.01) and NAD(P)H oxidase activity (10.3±1.0 vs. 5.2±1.6 MLU/min/mg protein, *P*<0.05) in the PVN caused by iWAT injection of capsaicin (Figure 6). AP5 alone also significantly attenuated the effects of the iWAT injection of capsaicin on the superoxide anion level and NAD(P)H oxidase activity in the PVN. However, CNQX alone only showed a tendency in attenuating the effects of capsaicin and the differences did not reach statistical significance.

### Effects of PVN microinjection of NMDA, AMPA, AP5 and CNQX

**Figure 7:**
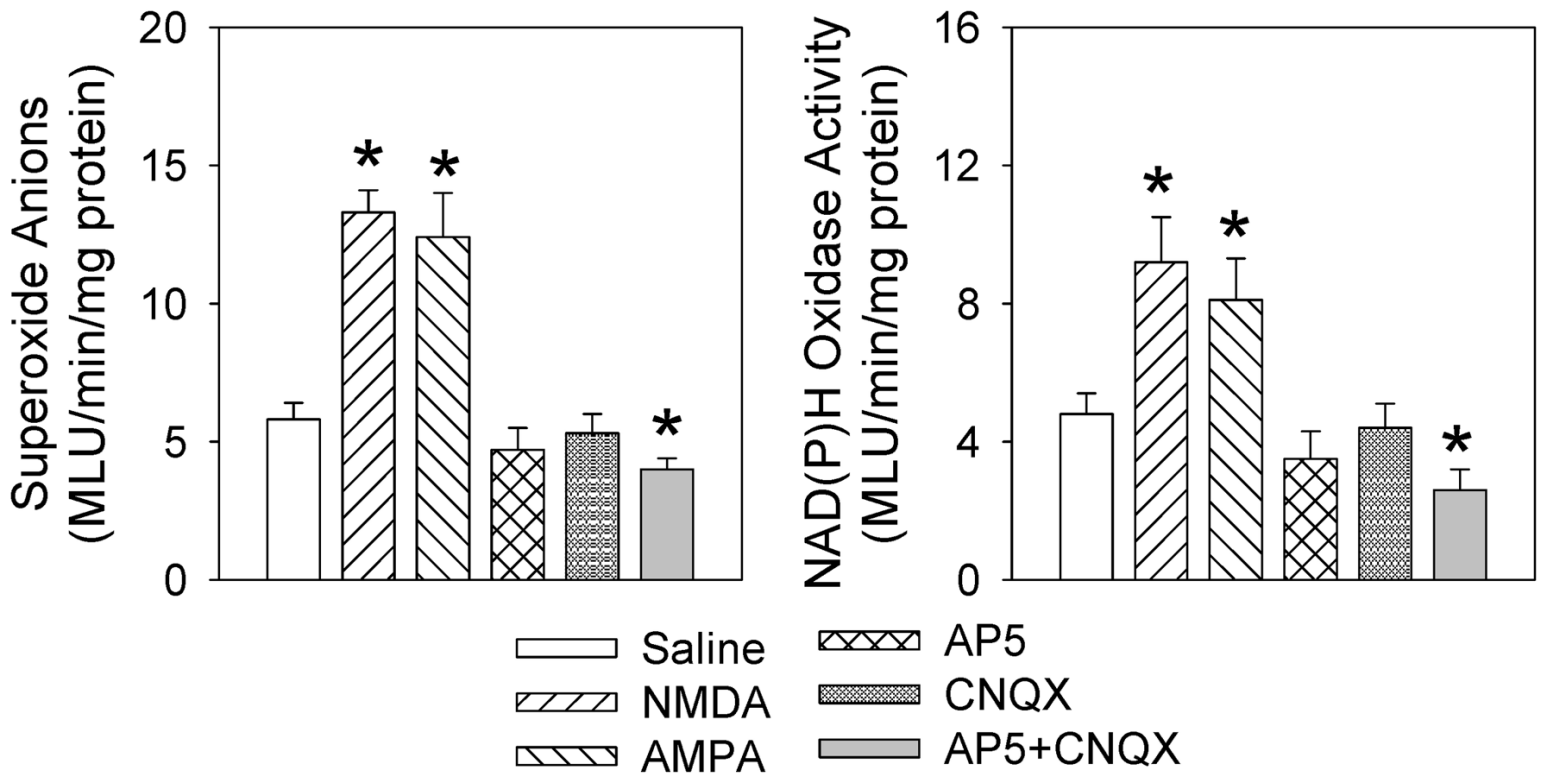
Effects of microinjection of NMDA, AMPA, AP5, CNQX or AP5 & CNQX into the PVN on superoxide anion level and NAD(P)H oxidase activity in the PVN. The measurements were made 15±SE. n = 6 for each group. *P<0.05 vs. Saline.

Microinjection of NMDAR agonist NMDA or non-NMDAR agonist AMPA into the PVN increased superoxide anion level and NAD(P)H oxidase activity in the PVN, while combined AP and CNQX reduced superoxide anion level (4.0±0.4 vs. 5.8±0.6 MLU/min/mg protein, *P*<0.05) and NAD(P)H oxidase activity (2.6±0.6 vs. 4.8±0.6 MLU/min/mg protein, *P*<0.05) in the PVN (Figure 7). Although the superoxide anion level and NAD(P)H oxidase activity in the PVN tended to be lowered after the PVN microinjection of AP5 or CNQX, the differences did not reach statistical significance.

## Discussion

Chemical stimulation of WAT afferents elicited AAR and resulted in sympathetic activation and pressor response, which were prevented by the lesion of bilateral PVN with kainic acid [28]. WAT injection of capsaicin to stimulate the WAT afferents increased c-fos expression in the PVN, and inhibition of PVN neurons with bilateral PVN perfusion of lidocaine abolished the AAR [33]. Combined blockade of NMDAR and non-NMDAR in the PVN interrupted the AAR [7]. After injection of herpes simplex virus-1 (HSV-1, an anterograde trans-neuronal viral tract tracer) into the WAT, the HSV-1-infected cells were found in the PVN [29]. These results indicate that PVN is an important component for the AAR. The primary new findings in the present study are that NAD(P)H oxidase-derived superoxide anions in the PVN modulates AAR, and ionotropic glutamate receptors in the PVN is involved in the AAR-induced production of superoxide anions in the PVN.

PEG-SOD is widely used to scavenge the intracellular superoxide anions and has a longer circulatory half-life than native SOD [17], [19]. It can rapidly penetrate into cells, whereas SOD has limited cellular penetration [2]. Tempol is another kind of membrane-permeable superoxide anion scavenger [36]. NAD(P)H oxidase is one of major origins of the superoxide anions [4]. In the present study, PVN microinjection of PEG-SOD, tempol or NAD(P)H oxidase inhibitor decreased baseline RSNA and MAP, and attenuated the AAR, indicating the importance of superoxide anions in the PVN in modulating the AAR, and NAD(P)H oxidase is a major source of AAR-induced superoxide anion production in the PVN. The results were supported by the findings that injection of capsaicin into the iWAT to induce AAR increased superoxide anion level and NAD(P)H oxidase activity in the PVN. Microinjection of PEG-SOD, tempol or apocynin into the PVN caused a slight decrease in baseline RSNA or baseline MAP, suggesting that endogenous superoxide anions in the PVN contribute a little to the tonic sympathetic outflow and blood pressure. On the other hand, PVN pretreatment with PEG-SOD, tempol or apocynin greatly attenuated the AAR, suggesting superoxide anions in the PVN play an important role in modulating AAR. It is noted that the AAR was induced by exogenous chemical stimulation of iWAT with capsaicin in the present study. The AAR caused by WAT afferent activity may be weaker in physiological state than that in the experimental stimulation state.

It was reported that neurons in culture and in mouse hippocampus responded to NMDA with a rapid increase superoxide production, followed by neuronal death. These events were blocked by the NAD(P)H oxidase inhibitor apocynin [3]. Glutamate induced production of reactive oxygen species in cultured forebrain neurons following NMDA receptor activation [25]. Abundant terminals with high glutamate immunoreactivity were found in the PVN [20]. Recently, we found that bilateral PVN microinjection of either NMDAR antagonist AP5 or non-NMDAR antagonist CNQX into the PVN attenuated the AAR, and combined AP5 and CNQX in the PVN abolished the AAR. Activation of NMDAR with NMDA potentiated the AAR, which was abolished by the pretreatment with AP5. Activation of AMPA receptors (AMPAR, a subtype of non-NMDAR) with AMPA also potentiated the AAR, which was abolished by pretreatment with CNQX. These results indicate that both NMDAR and non-NMDAR in the PVN contribute to the AAR, and most probably glutamate in the PVN mediates the AAR via activating both NMDAR and non-NMDAR in the PVN [7]. An interesting question is whether superoxide anions in the PVN are involved in the effects of NMDAR and non-NMDAR activation on the RSNA and blood pressure.

In the present study, we found that microinjection of combined AP5 and CNQX into the PVN blocked the responses of superoxide anions and NAD(P)H oxidase activity in the PVN to the iWAT injection of capsaicin. Microinjection of NMDA or AMPA into the PVN increased, but combined AP5 and CNQX reduced superoxide anion level and NAD(P)H oxidase activity in the PVN. These results indicate that the iWAT stimulation-induced activation of ionotropic glutamate receptors including NMDAR and non-NMDAR in the PVN contributes to the increased NAD(P)H oxidase activity and superoxide anion level in the PVN, which in turn causes sympathetic activation and pressor response. However, it seems that scavenging the superoxide anions in the PVN could not completely abolished the AAR. We excluded a possibility that the dose of PEG-SOD (5 units) or tempol (20 nmol) used in this study is not high enough to abolish the AAR because microinjection of 2 units of PEG-SOD or 20 nmol of tempol into the PVN completely abolished cardiac sympathetic afferent reflex (CSAR) [13], [37]. It is probable that some other downstream signal pathways besides superoxide anions may mediate the effects of NMDAR and non-NMDAR activation on the RSNA and blood pressure. It is noted that several signal molecules such as angiotensin II and endothelin-1 in the PVN are involved in the activation of NAD(P)H oxidase and the production of superoxide anions, and thereby causes sympathetic activation and pressor response [5], [13]. Angiotensin converting enzyme 2 (ACE2) gene therapy to the PVN reduced angiotensin II-mediated increase in NAD(P)H oxidase activity and normalized cardiac dysautonomia in ACE2(-/y) mice [32]. Whether these signal molecules are involved in the AAR-induced production of superoxide anions in the PVN is still unknown. In addition, reactive oxygen species in the RVLM enhance glutamatergic excitatory inputs and attenuate GABAergic inhibitory inputs to the RVLM, thereby increasing sympathoexcitatory input to the RVLM from the PVN in spontaneously hypertensive rats [23]. Whether superoxide anions in the PVN enhance glutamatergic excitatory inputs and attenuate GABAergic inhibitory inputs to the PVN merits further investigation.

In conclusion, scavenging superoxide anions or inhibiting the NAD(P)H oxidase activity in the PVN decreased baseline RSNA and MAP, and attenuated AAR. Chemical stimulation of WAT to induce AAR increased superoxide anion level and NAD(P)H oxidase activity in the PVN, which was abolished by the PVN pretreatment with combined NMDAR and non-NMDAR antagonists. Activation of NMDAR or non-NMDAR increased superoxide anion level and NAD(P)H oxidase activity in the PVN. These results indicate that NAD(P)H oxidase-derived superoxide anions in the PVN modulate AAR. Activation of ionotropic glutamate receptors in the PVN is involved in the AAR-induced production of superoxide anions in the PVN.
